# Association of chronic kidney disease with all-cause mortality in patients hospitalized for atrial fibrillation and impact of clinical and socioeconomic factors on this association

**DOI:** 10.3389/fcvm.2022.945106

**Published:** 2022-11-24

**Authors:** Min-qiang Bao, Gui-jun Shu, Chuan-jin Chen, Yi-nong Chen, Jie Wang, Yu Wang

**Affiliations:** ^1^Department of Neurology, The First Affiliated Hospital of Anhui Medical University, Hefei, China; ^2^Department of Neurology, Xuancheng People’s Hospital, Xuancheng, China; ^3^Department of Oncology, Xuancheng People’s Hospital, Xuancheng, China; ^4^Department of Medical Record Management, Xuancheng People’s Hospital, Xuancheng, China

**Keywords:** estimated glomerular filtration rate (eGFR), chronic kidney disease (CKD), atrial fibrillation (AF), socioeconomic status (SES), all-cause mortality

## Abstract

**Background:**

Atrial fibrillation (AF) and chronic kidney disease (CKD) often co-occur, and many of the same clinical factors and indicators of socioeconomic status (SES) are associated with both diseases. The effect of the estimated glomerular filtration rate (eGFR) on all-cause mortality in AF patients and the impact of SES on this relationship are uncertain.

**Materials and methods:**

This retrospective study examined 968 patients who were admitted for AF. Patients were divided into four groups based on eGFR at admission: eGFR-0 (normal eGFR) to eGFR-3 (severely decreased eGFR). The primary outcome was all-cause mortality. Cox regression analysis was used to identify the effect of eGFR on mortality, and subgroup analyses to determine the impact of confounding factors.

**Results:**

A total of 337/968 patients (34.8%) died during follow-up. The average age was 73.70 ± 10.27 years and there were 522 males (53.9%). More than 39% of these patients had CKD (eGFR < 60 mL/min/1.73 m^2^), 319 patients with moderately decreased eGFR and 67 with severely decreased eGFR. After multivariate adjustment and relative to the eGFR-0 group, the risk for all-cause death was greater in the eGFR-2 group (HR = 2.416, 95% CI = 1.366–4.272, *p* = 0.002) and the eGFR-3 group (HR = 4.752, 95% CI = 2.443–9.242, *p* < 0.00001), but not in the eGFR-1 group (*p* > 0.05). Subgroup analysis showed that moderately to severely decreased eGFR only had a significant effect on all-cause death in patients with low SES.

**Conclusion:**

Moderately to severely decreased eGFR in AF patients was independently associated with increased risk of all-cause mortality, especially in those with lower SES.

## Introduction

Chronic kidney disease (CKD) is a major public health problem that has many comorbidities and risk factors, such as diabetes, hypertension, and cardiovascular disease (1, 2). Many previous studies reported that low socioeconomic status (SES), as indicated by less education (3), low income (4), poverty (5), neighborhood deprivation (6), and other factors, are associated with disease progression and mortality in CKD patients. Thus, studies that aim to prevent mortality from CKD should consider SES as well as clinical indicators of kidney function.

Atrial fibrillation (AF) is the most common cardiac tachyarrhythmia, and several studies concluded that its prevalence will increase in the coming decades (7, 8). This increase is attributable to the aging population and the increased survival rates following myocardial infarction (9–11) and heart failure (12–14). The incidence rate of AF-related morbidity and mortality is also expected to increase over time, as will the conventional risk factors for AF, such as diabetes, hypertension, and cardiovascular disease (15–17). Factors related to SES, such as education, income, occupation, and characteristics of the community, could also potentially affect AF, but studies of these topics have had inconsistent results. For example, Kargoli et al. studied AF patients from New York City and showed that a lower SES predicted higher mortality after controlling for co-morbidities (18). In contrast, a national survey of the prevalence of AF in Scotland found that it had an inverse association with SES (19). AF may also be considered an epidemic that presents major socioeconomic challenges and is a public health issue at the global level (20, 21).

A population analysis showed that renal impairment affected 40 to 50% of patients with AF (22, 23). There is a reciprocal relationship between AF and CKD, in that CKD increases the risk of incident AF and AF increases the risk for the development and progression of CKD (24, 25). On the other hand, Ananthapanyasut et al. found a high prevalence of AF in patients with CKD who were not receiving dialysis, but estimated glomerular filtration rate (eGFR) did not correlate with the presence of AF in their population (22). The coexistence of AF and CKD can worsen the prognosis of each disease (26–28), and there seems to be an independent relationship between CKD and the risk of AF (28–31). Furthermore, although many studies have examined the effects of SES on CKD and on AF, few studies have reported the interrelationships of CKD, AF, and SES. Our aim was to assess the effect of CKD status on AF using data from a hospital electronic medical record system and to determine how this effect differs in patients with different SES.

## Materials and methods

### Study population

This single center retrospective cohort study evaluated the prognostic value of eGFR in patients with AF, and how this varied in different SES groups. From April 2017 to March 2019, patients aged 18 years or older were included if they were admitted to our institution with an ICD-10 code for AF (I48.x01, I48.x00, or I48.x01 × 022) based on review of the electronic medical record system. Finally, the records of 968 participants were retrieved and anonymized prior to analysis. According to China’s “Ethical Review Approaches for Biomedical Research Involving Humans” 2016, Article 39(1) (32), informed consent was not required for this retrospective study. The study was approved by the Institutional Ethics Committee of Xuancheng People’s Hospital (Anhui, China).

### Clinical outcomes

The primary outcome was all-cause mortality, and was evaluated by annual telephone follow-up and review of medical records. Subsequently, the date of death was confirmed through the civil affairs system or a death certificate from the medical records. Patients who were lost to follow-up were censored at the date of last contact. All patients were followed up until December 30, 2021.

### Covariates

Name, gender, age, hospitalization time, medical insurance category, and information on smoking, drinking, occupation, education, and medical history (coronary artery disease, congestive heart failure, hypertension, hyperlipidemia, hyperuricemia, diabetes, and other conditions) were collected from the electronic medical records and medical insurance system. Each medical condition was defined by the presence of a corresponding ICD-10 diagnostic code in the records. Patient outcome and time of death were determined through telephone follow-up, a death certificate, and records in the civil affairs system.

The estimated glomerular filtration rate (eGFR) was calculated using the Modification of Diet in Renal Disease (MDRD) equation for Chinese patients (33). Then, based on eGFR at admission, we defined CKD as an eGFR below 60 mL/min/1.73 m^2^, and stratified all patients into four groups.

eGFR-0: normal eGFR (≥ 90 mL/min/1.73 m^2^);
eGFR-1: mildly decreased eGFR (89–60 mL/min/1.73 m^2^);
eGFR-2: moderately decreased eGFR (59–30 mL/min/1.73 m^2^);
eGFR-3: severely decreased eGFR (< 30 mL/min/1.73 m^2^).

Coronary heart disease was ascertained by self-reported history of myocardial infarction, coronary artery bypass grafting, coronary angioplasty, or stenting, or if a patient had evidence of prior myocardial infarction in the baseline ECG, received an intervention, or had a coronary artery bypass graft. Hypertension was diagnosed when the blood pressure was 140/90 mmHg or more or if a patient used an antihypertensive agent. Diabetes mellitus was diagnosed when the fasting plasma glucose was 7.0 mmol/L or more, the random plasma glucose was 11.1 mmol/L or more, or a patient used a hypoglycemic drug. A lipid disorder was diagnosed when the total cholesterol was 5.7 mmol/L or more or when the LDL was 3.6 mmol/L or more. Hyperuricemia was diagnosed when the uric acid was 420 μmol/L or more or if a patient used a drug to control hyperuricemia. Education was categorized as not beyond the primary level or above junior high school. Medical insurance was divided as employee medical insurance or new rural cooperative medical insurance. Smoking status and drinking status were defined as current user or non-user (which included never used or used in the past but quit more than 1 year previously). The above data were used to calculate the CHA_2_DS_2_-VASc score (34) (1 point each for congestive heart failure, hypertension, diabetes, vascular disease, age 65–74 years, and female sex; and 2 points each for previous stroke/TIA/thromboembolism and age of 75 years or more).

### Statistical analysis

Categorical variables were presented as percentages and frequencies, and continuous variables as means ± standard deviations. Kaplan-Meier cumulative mortality curves were used to assess all-cause mortality, and curves for patients in different eGFR groups (see above) were compared using the log-rank test.

A multivariate Cox proportional hazard model was used to identify the independent effect of different variables on all-cause mortality, and subgroup analyses were used to determine whether the effect of eGFR was increased or decreased by different confounding factors. Models were adjusted for age group, sex, occupation, education, medical insurance, and other prognostic variables, including hypertension, diabetes, hyperuricemia, hyperlipidemia, coronary heart disease, heart failure, receipt of treatment (antiplatelet, anticoagulant, or lipid regulating drug), and history of smoking or drinking. These variables were selected based on clinical relevance and previous research (35). The results are reported as hazard ratios (HRs) with 95% confidence intervals (CIs) and *P* values. All analyses were performed using SPSS version 24 and a *P* value less than 0.05 was considered statistically significant.

## Results

**FIGURE 1:**
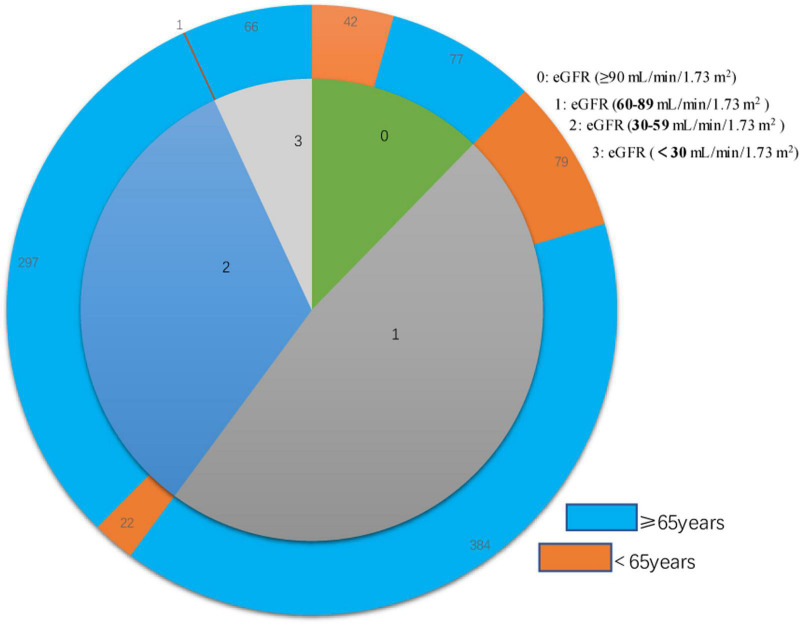
Double layer pie chart showing the numbers of patients who were less than 65 years-old and 65 years-old or more (outer circle) in the eGFR-0, eGFR-1, eGFR-2, and eGFR-3 groups (inner circle). Estimated glomerular filtration rate (eGFR) groups are defined in Table 1.

We examined 968 patients who were admitted to our institution for AF from April 2017 to March 2019 (Table 1). A total of 337 (34.8%) of these patients were deceased at the time of the last follow-up on December 30, 2021. The average age was 73.70 ± 10.27 years, and there were 522 males (53.9%) and 446 females (46.1%). The mean eGFR was 70.1 ± 26.9 mL/min/1.73 m^2^, and more than 39% of all patients had CKD (eGFR < 60 mL/min/1.73 m^2^). An indicated in a double-layer pie chart (Figure 1), there were 119 cases in the eGFR-0 group (eGFR > 90 mL/min/1.73 m^2^), 463 in the eGFR-1 group (eGFR = 60–89 mL/min/1.73 m^2^), 319 in the eGFR-2 group (eGFR = 30–59 mL/min/1.73 m^2^), and 67 in the eGFR-3 group (eGFR < 30 mL/min/1.73 m^2^).

**TABLE 1:** Baseline characteristics of patients in different estimated glomerular filtration rate (eGFR) groups who were admitted for atrial fibrillation.

Variable	eGFR group*				*P*
	eGFR-0 (*n* = 119)	eGFR-1 (*n* = 463)	eGFR-2 (*n* = 319)	eGFR-3 (*n* = 67)	
Age, years ± SD	66.40 ± 13.28	72.39 ± 9.71	76.96 ± 8.17	80.15 ± 6.90	**< 0.001**
Sex					**< 0.001**
Male	76	275	140	31	
Female	43	188	179	36	
Age, n					**< 0.001**
< 65 years	42	79	22	1	
≥ 65 years	77	384	297	66	
Medical insurance, n					0.152
Rural cooperative	90	355	261	57	
Employee-based	29	108	58	10	
Occupation status, n					0.067
Low	93	371	276	57	
High	26	92	43	10	
Education, n					**< 0.001**
<primary school	90	350	275	61	
>junior high school	29	113	44	6	
Hypertension, n					**< 0.001**
Yes	43	224	176	44	
No	76	239	143	23	
Diabetes, n					0.245
Yes	15	50	38	13	
No	104	413	281	54	
Hyperlipidemia, n					0.960
Yes	13	44	30	7	
No	106	419	289	60	
Hyperuricemia, n					**< 0.001**
Yes	9	78	130	41	
No	110	385	189	26	
Antiplatelet, n					0.437
Yes	66	273	200	37	
No	53	190	119	30	
Anticoagulant, n					**0.002**
Yes	29	122	58	6	
No	90	341	261	61	
Statin, n					0.067
Yes	63	244	157	24	
No	56	219	162	43	
Smoker, n					0.190
Yes	15	61	27	6	
No	104	402	292	61	
Drinker, n					**0.003**
Yes	18	54	20	2	
No	101	409	299	65	
CHF stage, n					**< 0.001**
1	74	228	108	13	
2	19	103	57	10	
3	21	109	115	31	
4	5	23	39	13	
CAD, n					**< 0.001**
Yes	24	156	141	34	
No	95	307	178	33	
CHA_2_DS_2_-VASc, mean ± SD	2.92 ± 1.73	3.55 ± 1.60	4.16 ± 1.52	4.55 ± 1.33	**< 0.001**

Anticoagulant, warfarin or non-vitamin K oral anticoagulant; Antiplatelet, aspirin or clopidogrel; CAD, coronary artery disease; CHF, congestive heart failure; eGFR, estimated glomerular filtration rate. *eGFR groups: eGFR-0, normal (≥ 90 mL/min/1.73 m^2^); eGFR-1, mildly decreased (89–60 mL/min/1.73 m^2^); eGFR-2, moderately decreased (59–30 mL/min/1.73 m^2^); eGFR-3, severely decreased (< 30 mL/min/1.73 m^2^).

Patients with moderate to severely decreased eGFR were older and more likely to be female than those with slightly decreased or normal eGFR (Table 1). A decreased eGFR was associated with an increased prevalence of coronary heart disease, cardiac insufficiency, hyperuricemia, and hypertension. Similarly, a low eGFR was associated with a higher CHA_2_DS_2_-VASc score. Overall, the eGFR-2 and eGFR-3 groups had more comorbidities and were less likely to use anticoagulants. However, eGFR was unrelated to use of antiplatelet therapy or statin therapy, both of which gradually declined with the decline of eGFR. In addition, patients with worse renal function were more likely to have rural cooperative medical insurance, a lower occupational status, and less education.

**FIGURE 2:**
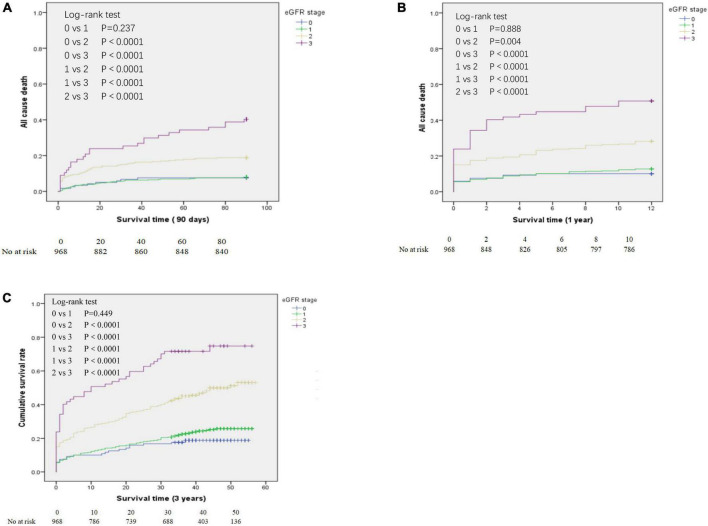
Kaplan-Meier analysis of all-cause mortality for patients in the different estimated glomerular filtration rate (eGFR) groups who survived at least 90 days **(A)**, at least 1 year **(B)**, and at least 3 years **(C)**. eGFR groups are defined in Table 1.

We determined the all-cause mortality rates of the different eGFR groups using Kaplan-Meier analysis and the log-rank test, with separate analyses for patients who survived at least 90 days (Figure 2A), at least 1 year (Figure 2B), and at least 3 years (Figure 2C). For patients who survived 90 days, 1 year, and 3 years, pair-wise comparisons indicated no significant differences in survival of the eGFR-0 and eGFR-1 groups (all *P* > 0.05). However, all other pair-wise comparisons for patients who survived 90 days, 1 year, and 3 years (eGFR-0 *vs.* eGFR-2, eGFR-0 *vs.* eGFR-3, eGFR-1 *vs.* eGFR-2, eGFR-1 *vs.* eGFR-3, and eGFR-2 *vs.* eGFR-3) indicated statistically significant differences in all-cause mortality (all *P* < 0.0001).

We also performed a Cox proportional hazard model to compare survival times in the different eGFR groups after adjusting for multiple confounding factors (Table 2). Relative to the eGFR-0 group, the adjusted HR for all-cause death was 2.416 (95% CI: 1.366–4.272, *P* = 0.002) in eGFR-2 group and 4.752 (95% CI: 2.443–9.242, *P* < 0.0001) in the eGFR-3 group. However, there was no significant difference in all-cause mortality in the eGFR-0 and eGFR-1 groups (HR = 1.307, 95% CI: 0.749–2.281, *P* = 0.345).

**TABLE 2:** Univariable and multivariable analysis of factors associated with all-cause mortality in patients admitted for atrial fibrillation.

Variable*	Univariable analysis		Multivariable analysis	
	HR (95% CI)	*P*	HR (95% CI)	*P*
**eGFR group**				
eGFR-0				
eGFR-1	1.479 (0.854–2.559)	0.162	1.307 (0.749–2.281)	0.345
eGFR-2	3.260 (1.899–5.600)	**<0.001**	2.416 (1.366–4.272)	**0.002**
eGFR-3	7.403 (4.018–13.642)	**<0.001**	4.752 (2.443–9.242)	**<0.001**
**CHF stage**				
1				
2	1.076 (0.739–1.568)	0.702	0.929 (0.630–1.368)	0.708
3	1.742 (1.284–2.362)	**<0.001**	1.211 (0.875–1.674)	0.248
4	2.816 (1.875–4.230)	**<0.001**	1.662 (1.082–2.554)	**0.020**
Anticoagulant	0.436 (0.299–0.636)	**<0.001**	0.581 (0.362–0.934)	**0.025**
Antiplatelet	1.478 (1.123–1.947)	**0.005**	1.030 (0.712–1.492)	0.874
Hyperuricemia	1.616 (1.239–2.107)	**<0.001**	1.014 (0.755–1.363)	0.925
More education	0.440 (0.295–0.657)	**<0.001**	0.663 (0.405–1.084	0.101
High-status occupation	0.591 (0.403–0.867)	**0.007**	1.207 (0.613–2.377)	0.586
Empl. medical insurance	0.556 (0.387–0.799)	**0.002**	0.703 (0.387–1.279)	0.249
Age ≥ 65 years	3.868 (2.211–6.767)	**<0.001**	2.424 (1.350–4.353)	**0.003**

Reference groups were eGFR-0, CHF stage-1, no anticoagulant use, no antiplatelet use, no hyperuricemia, less education, low-status occupation, rural cooperative medical insurance, and age < 65 years.

We performed a subgroup analysis to determine whether different factors increased or decreased the effect of eGFR on all-cause mortality (Table 3). Stratification by sex indicated that relative to the eGFR-0 group, males in the eGFR-2 group and females in eGFR-2 and eGFR-3 groups had significantly increased all-cause mortality (all *P* < 0.05). Also relative to the eGFR-0 group, there was significantly greater all-cause mortality in the eGFR-2 and eGFR-3 groups for those who had low occupational status, had no education beyond primary school, had rural medical insurance, did not use an anti-coagulant, and did not use an antihypertensive drug (all *P* < 0.05). In contrast, a decreased eGFR had no significant effect on all-cause mortality in those who had a high occupational status, had education above junior high school, had employee medical insurance, used an anti-coagulant, and used an antihypertensive drug (all *P* > 0.05).

**TABLE 3:** Subgroup analyses of the association of all-cause mortality with eGFR in patients admitted for atrial fibrillation*,**.

Subgroup								
eGFR group	HR	*P*	95% CI		HR	*P*	95% CI	
	Male				Female			
**eGFR-0 (ref)**								
eGFR-1	1.184	0.633	0.592	2.367	1.530	0.385	0.587	3.991
eGFR-2	**2.385**	**0.019**	**1.153**	**4.932**	**2.619**	**0.049**	**1.006**	**6.819**
eGFR-3	1.460	0.477	0.515	4.144	**9.845**	**< 0.001**	**3.472**	**27.911**

	**Low-status job**				**High-status job**			

**eGFR-0 (ref)**								
eGFR-1	1.331	0.367	0.715	2.479	1.031	0.963	0.280	3.798
eGFR-2	**2.753**	**0.002**	**1.464**	**5.178**	1.131	0.880	0.226	5.658
eGFR-3	**5.264**	**< 0.001**	**2.549**	**10.870**	1.930	0.531	0.247	15.050

	**Less than primary school**				**More than junior high school**			

**eGFR-0 (ref)**								
eGFR-1	1.573	0.167	0.827	2.994	0.535	0.307	0.161	1.776
eGFR-2	**3.036**	**0.001**	**1.578**	**5.840**	0.904	0.886	0.225	3.622
eGFR-3	**6.087**	**< 0.001**	**2.911**	**12.726**	0.455	0.552	0.034	6.126

	**Rural cooperative med. insurance**				**Employee med. insurance**			

**eGFR-0 (ref)**								
eGFR-1	1.343	0.354	0.720	2.504	1.432	0.584	0.395	5.190
eGFR-2	**2.604**	**0.003**	**1.379**	**4.917**	2.333	0.253	0.546	9.961
eGFR-3	**5.002**	**< 0.001**	**2.424**	**10.321**	3.912	0.211	0.462	33.152

	**No anticoagulant**				**Anticoagulant**			

**eGFR-0 (ref)**								
eGFR-1	1.289	0.406	0.708	2.346	1.630	0.543	0.338	7.871
eGFR-2	**2.364**	**0.006**	**1.282**	**4.362**	2.763	0.216	0.552	13.834
eGFR-3	**4.775**	**< 0.001**	**2.340**	**9.745**	7.064	0.057	0.943	52.931

	**No antihypertensive**				**Antihypertensive**			

**eGFR-0 (ref)**								
eGFR-1	1.493	0.206	0.803	2.777	0.524	0.329	0.144	1.915
eGFR-2	**2.870**	**0.001**	**1.520**	**5.417**	0.975	0.970	0.262	3.626
eGFR-3	**5.130**	**< 0.001**	**2.436**	**10.800**	3.870	0.095	0.789	18.982

*Abbreviations and eGFR groups are defined in Table 1. **Models were adjusted for age group, sex, occupation, education, medical insurance, and other prognostic variables, including hypertension, diabetes, hyperuricemia, hyperlipidemia, coronary heart disease, heart failure, receipt of treatment (antiplatelet, anticoagulant, or lipid regulating drug), and history of smoking or drinking.

## Discussion

Our study demonstrated that moderately to severely decreased eGFR was an independent risk factor for all-cause mortality in patients admitted for AF. Moreover, the impact of moderately to severely decreased eGFR on all-cause mortality in these patients was increased for follow-up times of 90 days, 1 year, and 3 years. We also found that SES had an impact on the outcome of these patients. In particular, our subgroup analyses indicated that eGFR was significantly related to all-cause mortality in patients with low SES (low-status employment, less education, rural cooperative medical insurance, no use of an anticoagulant drug, no use of an anti-hypertensive drug), but this relationship was not significant in patients with high SES (high-status employment, more education, employee medical insurance, and those who used an anticoagulant or an anti-hypertensive drug).

Atrial fibrillation (AF) and CKD often coexist, and our AF patients had a high prevalence of chronic renal insufficiency. More specifically, patients with CKD (moderately to severely decreased eGFR) accounted for about 39% of our patients, similar to previous studies (36). Our results are also in line with the results of Shinya et al. (37), who found that patients with moderately to severely decreased eGFR were older and more likely to be female. There are several common and well-known risk factors CKD and AF, such as hypertension, diabetes, and obesity (38–42). CKD and AF are also associated with age-related diseases (43, 44). Thus older patients are more likely to have CKD and AF. It is generally believed each condition aggravates the other, and together they contribute to poor patient prognosis.

The adverse prognosis of patients with decreased renal function and AF is not simply reflected in the increased number and severity of complications, but also affects the choice and efficacy of treatments. For example, the risk of stroke and bleeding in these patients group increases gradually as renal function declines (45, 46), possibly making it inappropriate to administer an anticoagulant. However, some predictors of AF in the general population differ from those in patients with CKD (47). That is, some risk factors have attenuating effects on the relationship between AF and CKD, thereby emphasizing the importance of assessing the relationship between AF and CKD and clinical outcomes in different clinical settings.

We examined a cohort of patients hospitalized for AF. eGFR is usually used to evaluate the status of renal function (48) because it is an easily measured and comprehensive clinical index of renal function. We found that even after adjusting for many confounding factors, moderately to severely decreased eGFR remained an independent risk factor for all-cause mortality, consistent with many previous studies (29, 49). A recent study based on the GARFIELD-AF Registry (37) examined the impact of different stages of chronic renal insufficiency on the prognosis of patients with AF. This study indicated that moderate to severe CKD was an independent predictor of all-cause death within 1 year of the diagnosis of AF. We performed subgroup analysis to examine the effect of follow-up time on outcome, and our results suggested that moderately to severely decreased eGFR had similar effects on all-cause mortality at 90 days, 1 year, and 3 years.

The global incidence rate of AF has increased significantly over time, as have the incidence rates of diseases traditionally associated with AF (hypertension, diabetes, cardiovascular diseases, obesity, and metabolic syndrome) (39, 40). Income, education level, living conditions, and health awareness are indicators of SES, and they also impact the prognosis of patients with AF. Numerous studies demonstrated that a lower SES was associated with increased mortality in patients with AF (18, 50, 51). In addition, a large multi-ethnic cohort study found that people who did not know they had AF had an increased risk of mortality compared with those who did know they had AF (52).

However, some studies reported contrary results. For example, a national survey of AF in Scotland found that its prevalence decreased with increasing socioeconomic deprivation (19). We found that low SES was associated with an increased risk of death from AF, but not after adjustment for other risk factors. Furthermore, we found that moderately to severely decreased eGFR had a more significant effect on all-cause death in people with lower SES. A previous study in Sweden demonstrated that community deprivation and socio-economic differences were not independent risk factors for hospitalization due to AF (53). However, these researchers suggested that SES may indirectly increase the risk of AF and poor prognosis due to its affect on comorbidities that are associated with AF (53). This suggests that when a patient presents with CKD, AF, and low SES, clinicians should focus on preventive measures and seek to increase the access to additional medical resources that can improve overall prognosis.

We found that the CHA_2_DS_2_-VASc score gradually increased with the deterioration of kidney function, but these patients also had decreased use of anticoagulants. This may be because clinicians are reluctant to prescribe anticoagulants for these patients because of their increased risk of massive bleeding. Anticoagulation therapy remains the cornerstone of the treatment for atrial fibrillation (54). However, when AF coexists with renal insufficiency, the patient also has a high risk of death and massive bleeding (55). Additionally, no randomized controlled trials have yet examined the effect of oral anticoagulants in patients with renal insufficiency. In general, data on how to best treat AF in patients with CKD are extremely limited, and the most appropriate treatment is likely to be complex. Our study showed that anticoagulant therapy had an independent protective effect on all-cause death in patients with AF and CKD. Although our evidence does not prove the benefit of anticoagulant use in these patients, it supports the results of Anders et al. (56), who showed that warfarin treatment provided a net clinical benefit in patients with chronic renal insufficiency.

There were several limitations in this study. First, our study was a single-center retrospective analysis, so there is a possibility of selection bias. Second, despite our consideration of routine prognostic factors, it is likely there were additional unknown confounders that we did not consider, especially some indicators of SES (household income, neighborhood, etc.). Finally, our study had relatively small sample size and short follow-up time. These challenges need to be addressed in a study that uses a larger sample size and accounts for more potential confounders.

## Conclusion

It is well-known that the prevalence of CKD increases with age. We found that a moderately to severely decreased eGFR was an independent risk factor for all-cause death in patients hospitalized for AF, especially in those with low SES. Thus, the main clinical relevance of this study is that clinicians should pay special attention to AF patients who present with modifiable factors that correlate with low SES. In addition, healthcare systems should consider the implementation of policies that improve disease prevention and increase the access to medical resources by individuals with low SES, because these likely to improve the health status of these individuals.

## Data availability statement

The original contributions presented in this study are included in the article/supplementary material, further inquiries can be directed to the corresponding author.

## Ethics statement

The studies involving human participants were reviewed and approved by the Institutional Ethics Committee of Xuancheng People’s Hospital (Anhui, China). The ethics committee waived the requirement of written informed consent for participation.

## Author contributions

M-QB and YW designed/performed most of the investigation, data analysis, and wrote the manuscript. G-JS and C-JC sort out and manage case data. Y-NC and JW contributed to interpretation of the data and analyses. All authors have read and approved the manuscript.
